# Are High-Temperature Molten Salts Reactive with Excess Electrons? Case of ZnCl_2_

**DOI:** 10.1021/acs.jpcb.3c04210

**Published:** 2023-09-27

**Authors:** Hung H. Nguyen, Vyacheslav S. Bryantsev, Claudio J. Margulis

**Affiliations:** † Department of Chemistry, The University of Iowa, Iowa City, Iowa 52242, United States; ‡ Chemical Sciences Division, Oak Ridge National Laboratory, Oak Ridge, Tennessee 37831, United States

## Abstract

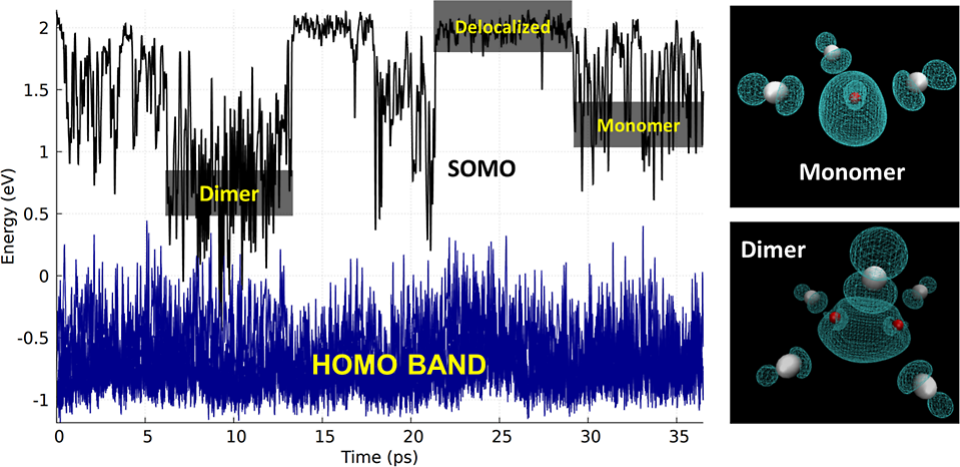

New and exciting frontiers for the generation of safe and renewable energy have brought attention to molten inorganic salts of fluorides and chlorides. This is because high-temperature molten salts can act both as coolants and liquid fuel in next-generation nuclear reactors. Whereas research from a few decades ago suggests that salts are mostly unreactive to radiation, recent experiments hint at the fact that electrons generated in such extreme environments can react with the melt and form new species including nanoparticles. Our study probes the fate of an excess electron in molten ZnCl_2_ using first-principles molecular dynamics calculations. We find that on the time scale accessible to our study, an excess electron can be found in one of three states; the lowest-energy state can be characterized as a covalent Zn_2_Cl_5_^•2–^ radical ion, the other two states are a solvated Zn^•+^ species (ZnCl_3_^•2–^) and a more delocalized species that still has some ZnCl_3_^•2–^ character. Since for each of these, the singly occupied molecular orbital (SOMO) where the excess charge resides has a distinct and well-separated energy, the different species can in principle be characterized by their own electronic spectra. The study also sheds light onto what is commonly understood as the spectrum of a transient radical species which can be from the SOMO onto higher energy states or from the melt to pair with the excess electron leaving a hole in the liquid.

## 1 Introduction

The existence of new models, prototypes, or studies^1−4^ on molten salt reactors that promise to deliver electricity with no carbon emissions has resulted in questions about the chemistries that can take place in ionic melts under extreme conditions of temperature and radiation. Even though early reports suggested a lack of reactivity under radiation conditions in certain ionic melts,^5^ more recent studies (see ref (6) and citations therein) put some of these conclusions into question. Hence, issues of interest include the formation of reactive species and their behavior at interfaces or in bulk, the possibility of the formation of insoluble metal particles in the melt, and the formation of volatiles such as F_2_ or Cl_2_. Radiolysis experiments on molten salts^6−8^ are perhaps the most useful tool to address such questions, but even these cannot alone provide a full detailed atomistic description of processes occurring in the melt. As an example, simulation work on electrons and holes on the colder relatives of the inorganic molten salts, the room-temperature ionic liquids (RTILs),^9−12^ have provided significant insight into the ultrafast dynamics observed in experiments where electrons are generated *via* optical excitation.^13^ Two types of simulation schemes are often used to study the fate of an electron or hole in the condensed phase. The first one assumes that no reactivity or chemistry can occur since the melt is considered as having no electronic states besides those of the excess electronic species.^14,15^ In this scheme, a semiclassical propagation approach is taken^14^ for the ions within the context of multiple electronic states of the one-electron system. The second approach, the one taken in this work, is that of first-principles molecular dynamics, where the possibly reactive multi-electron melt is considered in the presence of an excess charge and propagated classically on the Born–Oppenheimer surface;^9−12^ in this scheme, the system is always in the ground electronic state. Each of these approaches has its advantages and disadvantages, for example, the first approach is much better at studying the actual quantum dynamics of an electron that can be “hot” and have non-adiabatic behavior, whereas the second allows for the study of ground-state chemical reactivity in solution, which is our goal.

**Figure 1:**
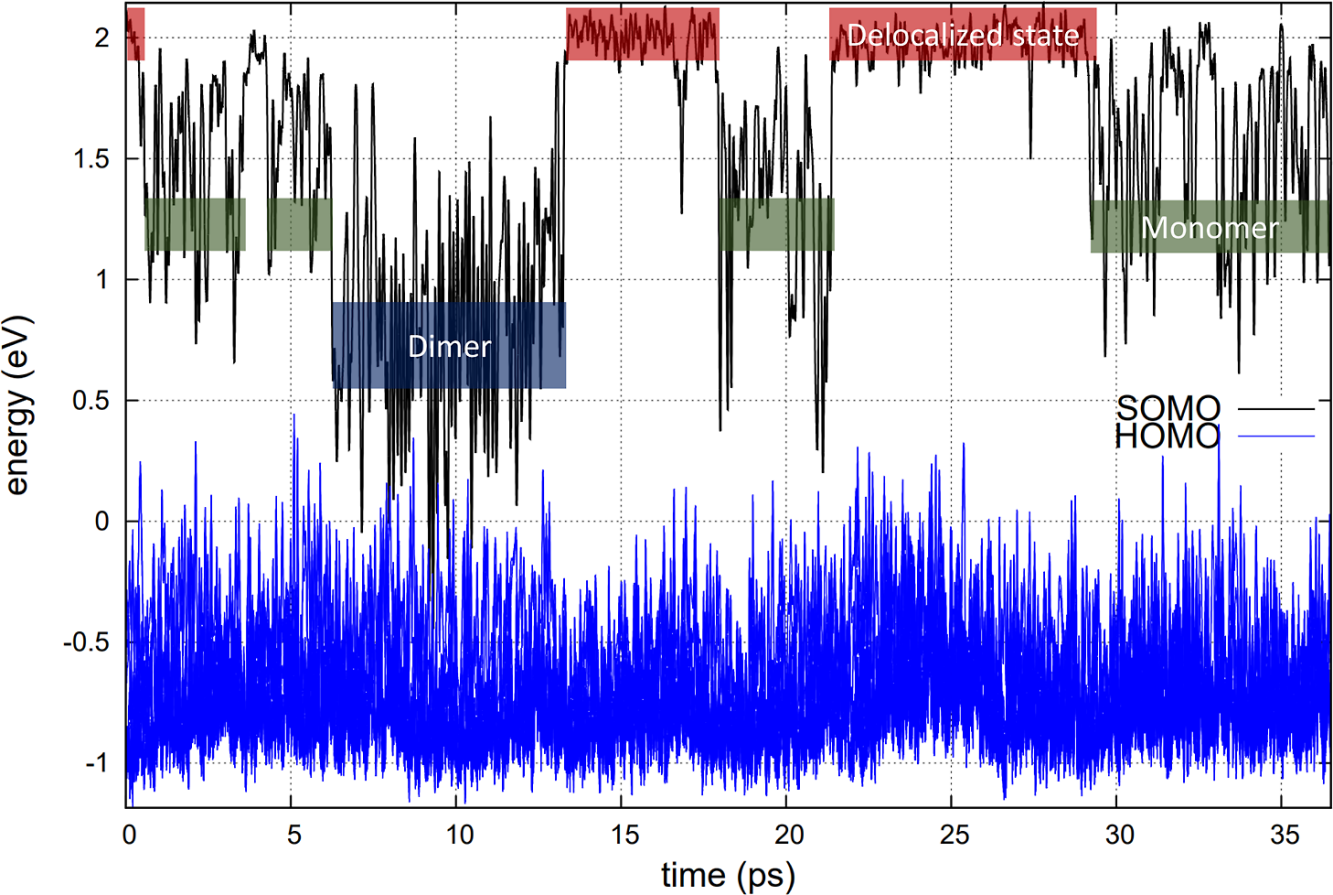
HOMO band (20 states) and SOMO state for ZnCl_2_ in the presence of an excess e^–^. Red, green, and blue boxes correspond to states that can be described as delocalized, as ZnCl_3_^•2–^ (monomeric zinc), and as Zn_2_Cl_5_^•2–^ (dimeric zinc), respectively.

Our study starts from the well-equilibrated liquid structure of molten ZnCl_2_ at 600.15 K that we recently published in ref (16) and that well reproduces the neutron structure function *S*(*q*). For ZnCl_2_, the 4-coordinated tetrahedral state of Zn^2+^ is dominant and the melt consists of extended networks of corner- and edge-sharing units^16,17^ (see, for example, Figures 1 and 8 in ref (16)); the majority of Zn^2+^ complexes within the network share one Cl^–^ counterion (corner sharing), but some units share two (edge sharing). This is as opposed to other divalent metal ion melts such as MgCl_2_ where multiple coordination states of Mg^2+^ are likely, including 4, 5, and 6, with 5 being the most popular.^16,18−20^ In the pre-equilibrated ZnCl_2_ system, an excess e^–^ is introduced and we follow its dynamics as a function of time. Because the system is now charged and a jellium approximation is introduced, we also run simulations in which the electron replaces one chloride ion, rendering the system neutral. Both studies yield results that are consistent, providing confidence in our analysis. Therefore, except when we want to highlight the effect of an anionic vacancy in the liquid, most of our discussion will be based on the system with an overall excess charge.

As will become clear in the Results and Discussion section, the electron can attach to one Zn^2+^ ion to form Zn^•+^ (perhaps better described as ZnCl_3_^•2–^, but sometimes ZnCl_4_^•3–^), it can bridge two Zn^2+^ ions and change their electronic structure to form the molecular species Zn_2_Cl_5_^•2–^ (sometimes Zn_2_Cl_6_^•3–^), or it can remain delocalized. The delocalized state still has one Zn^2+^ that carries more spin density and we could also call it Zn^•+^; however, the local spin density enhancement is much lower and energetics as well as local charges are very different when contrasted to the localized state. Hence, we prefer to think of these as two distinct species.

Each of the three species we have identified has a specific electronic spectrum, and each of the spectra can potentially have contributions that are from bonafide transitions of the excess e^–^ or from electrons in the melt into the singly occupied molecular orbital (SOMO) or higher energy states. What is interesting about this is that when radiolysis experiments identify a species such as Zn^•+^, they are not able to separate the transitions of that species (*i.e.*, the transitions that originate from SOMO) from those of the “solvent” into SOMO. Understanding this subtle difference can help us rationalize how such species may behave differently in different media with different band gaps. The fact that species such as Zn_2_Cl_5_^•2–^ can form in the melt begs the question of what other species can form when the flux of electrons is high enough that different radicals can interact (which is not the case in our study). We speculate that it is not unlikely that such interactions may lead to the formation of nanoparticles such as those discussed for Ni^2+^-doped ZnCl_2_ melts in ref (21).

## 2 Methods

All *ab initio* molecular dynamics (AIMD) simulations were carried out using the Quickstep module of the CP2K package^22,23^ at the Perdew–Burke–Ernzerhof (PBE)^24^ density functional level of theory combined with Grimme’s D3 dispersion correction.^25^ We used the MOLOPT-DZVP^26^ basis set combined with the Goedecker–Teter–Hutter (GTH)^27^ pseudopotentials, where for Zn the 1s^2^2s^2^2p^6^3s^2^3p^6^ electrons were considered part of the pseudopotential and for Cl the 1s^2^2s^2^2p^6^ were considered part of the pseudopotential. We used the orbital transformation method^28^ with the FULL_ALL preconditioner and the conjugate gradient minimizer. CUTOFF and REL_CUTOFF parameters were set to 600 and 60 Ry, respectively, and the EPS_SCF convergence was set to 10^–6^ for both inner and outer loops. The always stable predictor corrector extrapolation was used, with an extrapolation order of 4, and the parameter EPS_DEFAULT was set to 10^–12^. All simulations were run in the canonical ensemble (*NVT*) with a fixed box length of 23.738 Å. The temperature was fixed at 600.15 K using the Nosé–Hoover thermostat^29,30^ with a chain length of 3 and a time constant of 1.0 ps; the time step was 1 fs. To describe the excess e^–^, a negative charge was added to a simulation box derived from our prior work,^16^ resulting in a total charge of 1e^–^ and a multiplicity of 2. Our simulation in the presence of the excess e^–^ was 36.5 ps in duration and included 149 ZnCl_2_ formulas. For testing purposes, a separate trajectory was run in which an electron was added and a Cl^–^ ion was removed, yielding the system neutral. All calculations in the presence of the excess electron are spin-polarized (CP2K keyword UKS).

To better understand the distribution of the excess charge in our condensed phase system, we computed Bader charges as a function of time.^31,32^ In some cases, we also performed first-principles calculations in the gas phase. When doing so, we used the B3LYP^33−36^ functional together with the def2TZVP basis set^37^ as coded in the Gaussian 09 package.^38^

Whereas we can gain important information from our AIMD simulations using the PBE-D3 functional, to compute the spectrum of selected simulation frames associated with Zn_2_Cl_5_^•2–^, ZnCl_3_^•2–^, as well as the delocalized e^–^ state, we went beyond this level of theory and used the TDDFT technique for periodic systems implemented in CP2K using the Tamm–Dancoff approximation^39^ together with the PBE0 exchange–correlation functional^40^ and the Grimme dispersion correction.^25^ The CUTOFF_RADIUS for the exchange part of PBE0 was set to 4.0 Å. Auxiliary density matrices^41^ were also implemented to reduce the cost of calculating the Hartree Fock energy. To encompass excitation energies up to 4.5 eV, the number of computed excited states for each snapshot varied between 150 and 200.

## 3 Results and Discussion

Starting from the same equilibrated frame in the absence of an excess e^–^, we run AIMD simulations for two different scenarios. The first scenario (which is the one that we will be describing in this work unless we explicitly say otherwise) corresponds to an electron introduced in ZnCl_2_, a melt not pre-equilibrated to have an excess charge. In this case, the excess e^–^ has no obvious preferential location at time zero. The second scenario is one in which an anion was removed and an excess e^–^ was introduced. For the first scenario, the early subpicosecond regime is often described as the “dry electron”; this is in contrast to an electron in which the surrounding medium has had time to undergo structural relaxation in its presence and hence is called a “solvated electron”.

**Figure 2:**
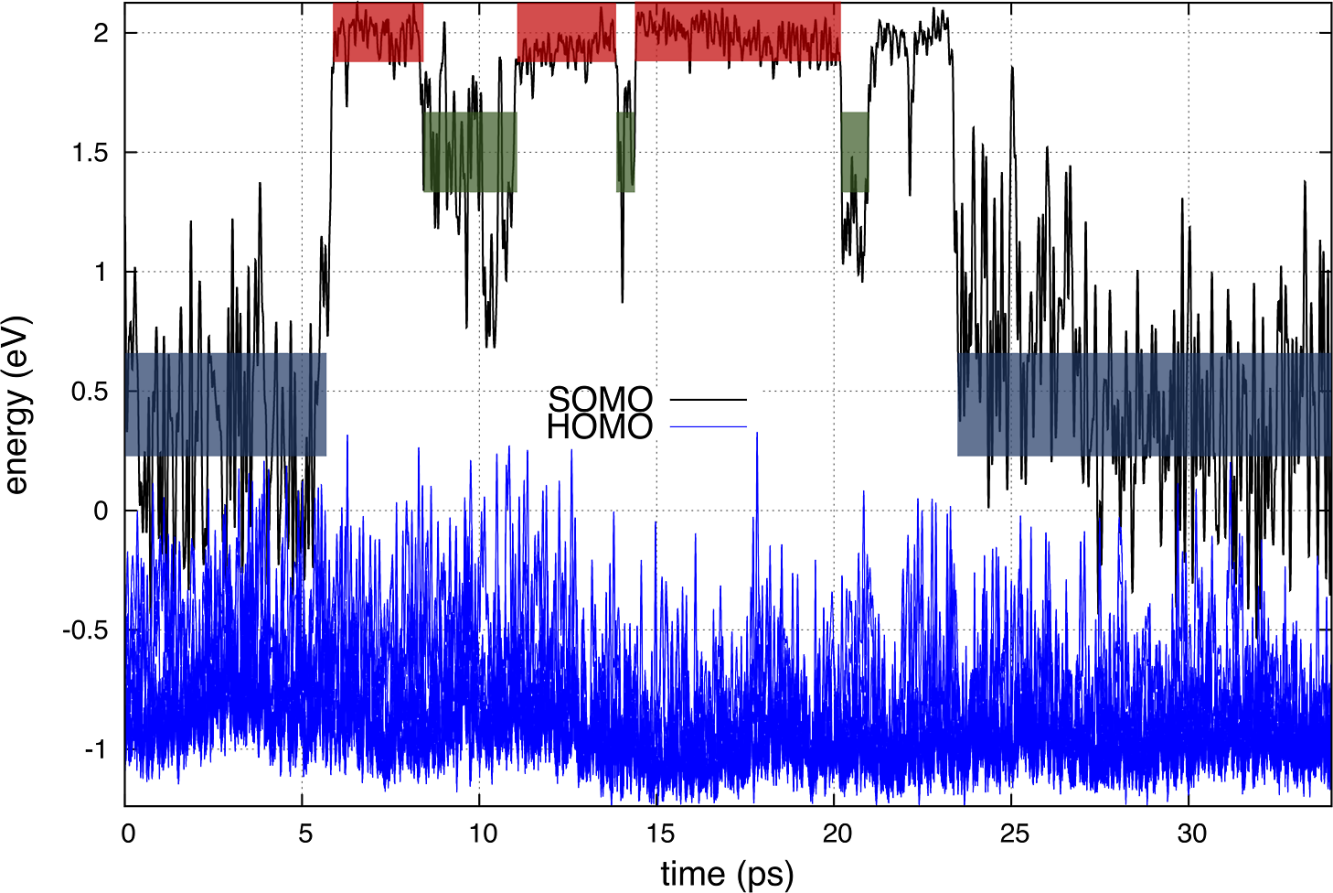
HOMO band (20 states) and SOMO state for ZnCl_2_ in the presence of an excess e^–^ in the case where a Cl^–^ ion has been removed at time zero. Just like in Figure 1, red, green, and blue boxes represent delocalized, monomeric, and dimeric states, respectively.

**Figure 3:**
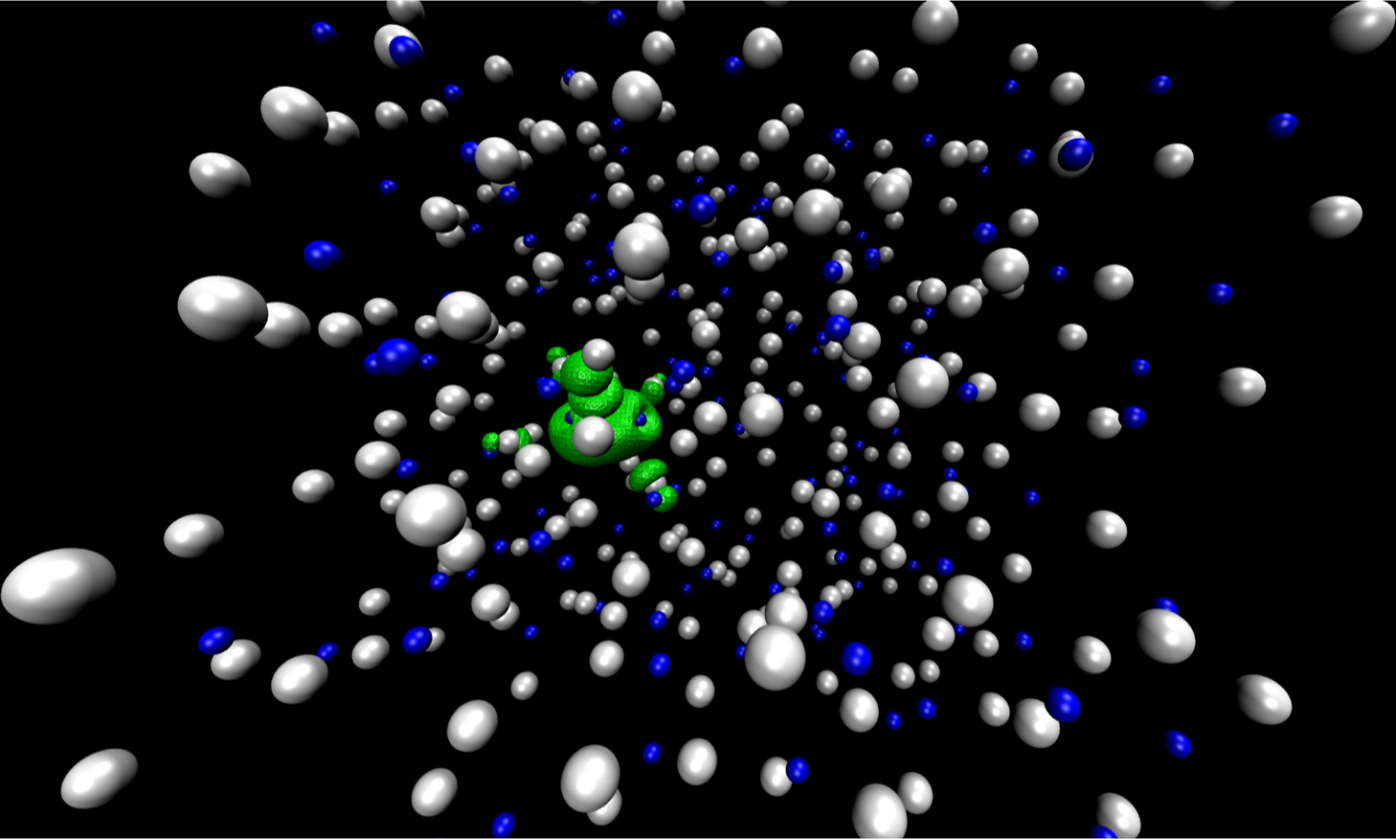
Simulation snapshot including the spin density computed using the PBE0-D3 method. Cl^–^ is in white, Zn^2+^ in blue, and the spin density, which in this case localizes on Zn_2_Cl_5_^•2–^ is in green (see also Figures 5 and 6).

Figure 1 shows, as a function of time, the evolution of SOMO and HOMO states. We see that after a short subpicosecond period in which the electron is delocalized (see initial red box in Figure 1), the SOMO energy quickly evolves into a second type of state (green box in Figure 1) that we associate with Zn^•+^ (or ZnCl_3_^•2–^). In our simulation, this state later changes into the lowest-energy SOMO state that can be associated with Zn_2_Cl_5_^•2–^. At later times, the Zn^•+^ state and the delocalized state reappear. Figure 1 should be contrasted with Figure 2, which is for the trajectory where a Cl^–^ was removed at time zero. In Figure 2, the same three SOMO energy states can be detected. However, in contrast to the first scenario, at time zero, the excess e^–^ has a pre-equilibrated location where the removed Cl^–^ ion used to be. This results in the excess e^–^ quickly localizing in the low-energy dimeric state. Figure 3 shows a typical snapshot of our system in which the excess electron is localized on a pair of Zn^2+^ ions forming Zn_2_Cl_5_^•2–^.

In Section 3.1, we describe in more detail the nature of the three species, including an analysis of charges, the projected density of states, and other characteristics; Section 3.2 discusses their distinct optical spectral features.

### 3.1 Nature of the Species

**Figure 4:**
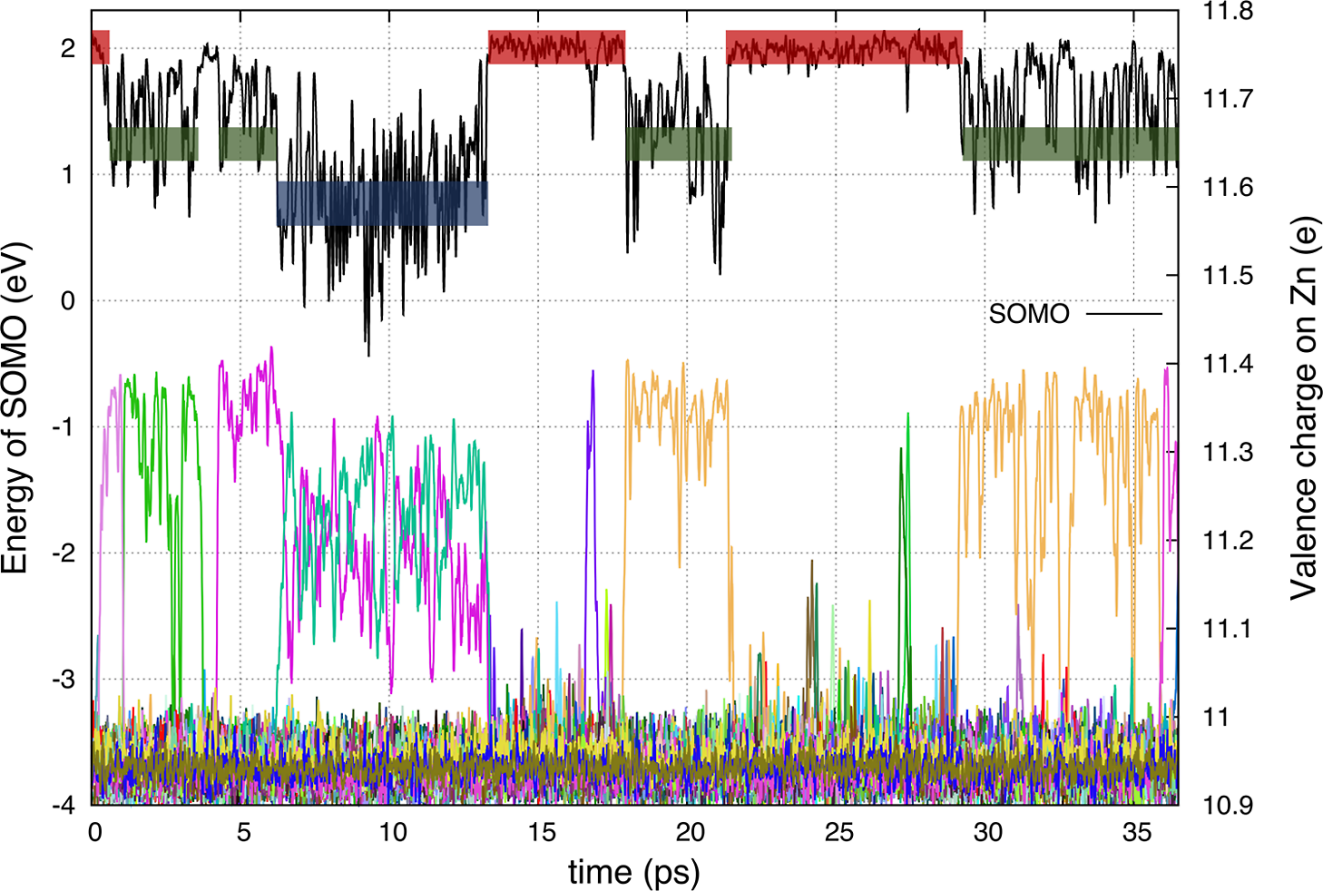
(Color lines) time-dependent Bader charges for the different Zn species (only electrons that are not part of the pseudopotential are considered) and (black line) energy of SOMO, all computed under the PBE-D3 approximation. Just like in Figure 1, red, green, and blue boxes represent delocalized, monomeric, and dimeric states, respectively.

Figure 4 shows what changes in ionic charge occur concomitant with changes in SOMO energy. For example, at a very short time in the subpicosecond regime, no Zn^2+^ ion has a dominant fraction of the excess electron charge, but soon after, the charge localizes on a single Zn^2+^ to form Zn^•+^.

Notice that up to about 7 ps, we see two charge transfer events resulting in three different ZnCl_3_^•2–^ radical ions. After this, in the regime up to about 13 ps, two different metal ions share the excess e^–^ charge, each with a fraction of it. This is one of the signatures for the existence of Zn_2_Cl_5_^•2–^. At later times, the charge becomes delocalized or transfers to a single metal ion, which we again characterize as the radical cation Zn^•+^. Notice that even in the delocalized state, there is still a metal species that often has more charge than the rest. Yet, we make a distinction between this state and ZnCl_3_^•2–^ because charges are very different, the SOMO energetics are very different and also the projected density of states and spectroscopy of these are very different (*vide infra*).

**Figure 5:**
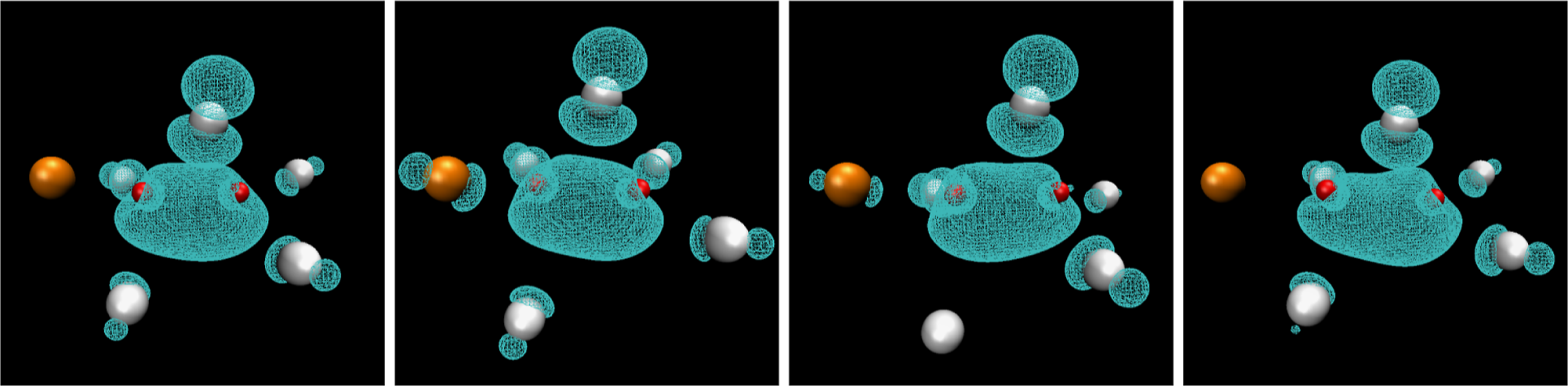
Structure of Zn_2_Cl_5_^•2–^ from different snapshots along our condensed phase simulation computed at the PBE0-D3 level of theory. These are the same snapshots used to compute the spectra for this species. The metal ion is depicted in red and Cl^–^ in white, except that there is one extra Cl^–^ ion in the first frame depicted in orange that is at bonding distance but does not share significant spin density. In subsequent frames, we see the white and orange Cl^–^ ions exchange roles, including a transition situation in which all Cl^–^ ions share significant spin density.

**Figure 6:**
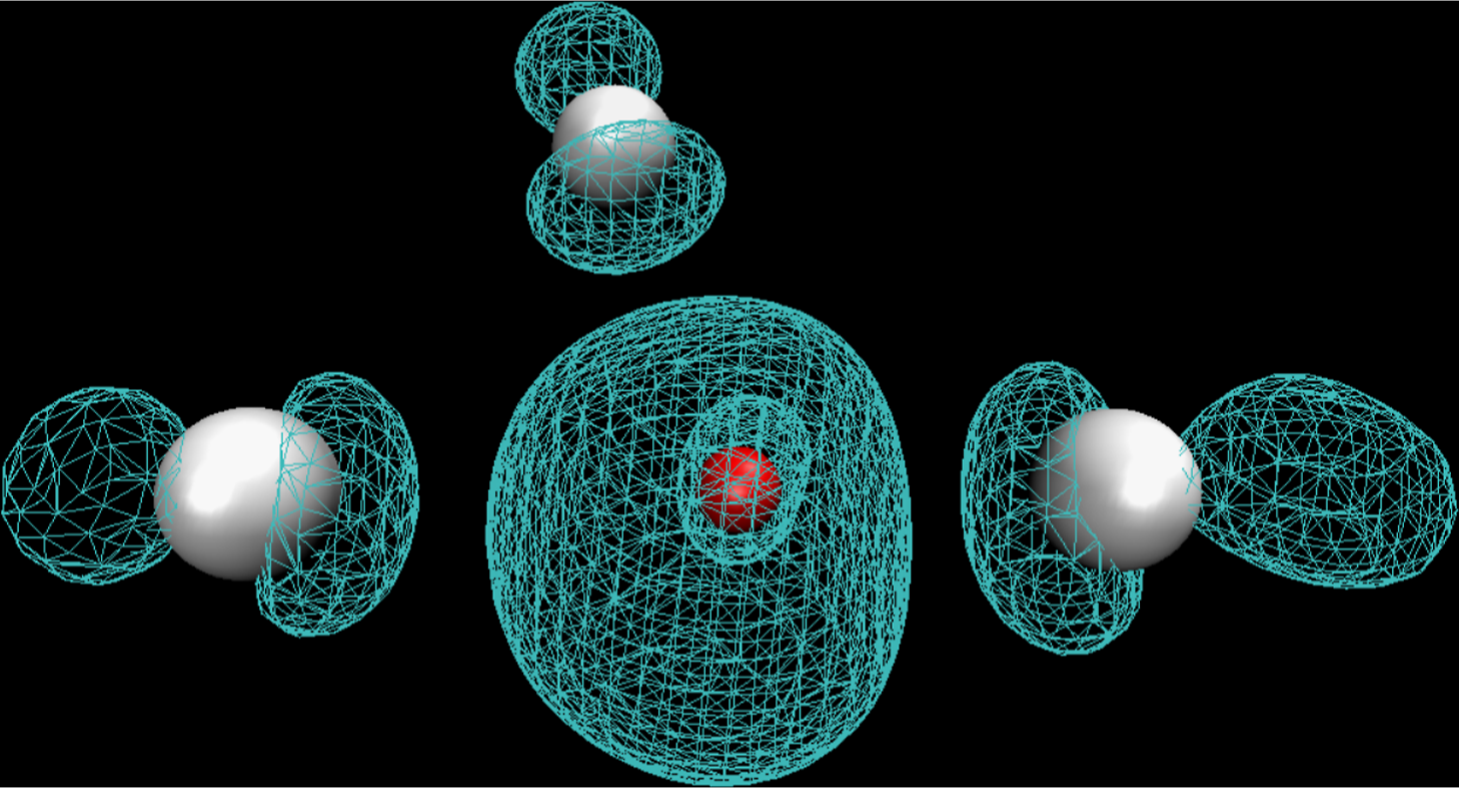
Typical structure of ZnCl_3_^•2–^ computed at the PBE0-D3 level of theory from a simulation snapshot. The metal ion is in red and the Cl^–^ ions are in white.

Figure 5 shows examples of Zn_2_Cl_5_^•2–^ along simulation in which the spin density is shared between metal centers; Figure 6 shows instead the structure of ZnCl_3_^•2–^. In Figure 5, we include an extra Cl^–^ to highlight that the anions in Zn_2_Cl_5_^•2–^ can quickly exchange.

**Figure 7:**
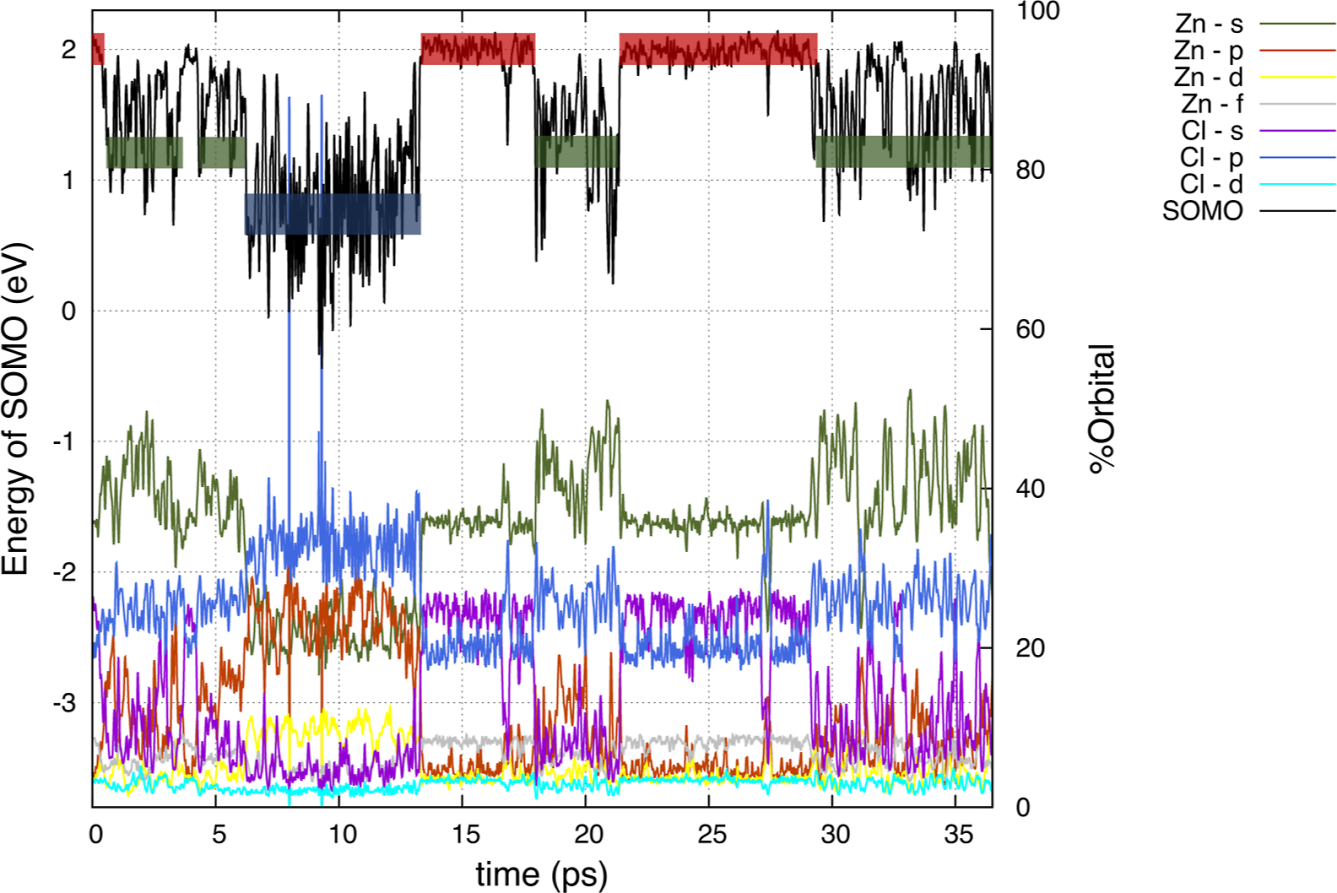
(Color lines) Time-dependent PDOS and (black line) energy of SOMO, all computed under the PBE-D3 approximation. Just like in Figure 1, red, green, and blue boxes represent delocalized, monomeric, and dimeric states, respectively.

The nature of bonding becomes even more clear when considering the projected density of states (PDOS) as a function of time, as depicted in Figure 7. It is important to look at this figure in combination with the box labels in Figure 1. Each time the system switches between the delocalized state, the monomeric state, and the dimeric molecular state, the pattern in the PDOS switches. But if we compare time segments for the same species, the pattern is always the same. In the monomeric Zn^•+^ state, the major contribution is from the S orbitals of the metal ion and the P orbitals of Cl^–^; all other contributions are much less significant. In the delocalized state, the S orbitals of the metal are important but both S and P orbitals of Cl^–^ contribute significantly. The most interesting situation occurs for Zn_2_Cl_5_^•2–^ where S and P orbitals of the metal appear to mix. In the gas phase, where an NBO analysis^42^ is easier to carry out, the resulting hybridization between metal centers is SP^2^; we note that the spin density in the condensed phase and the SOMO in the gas phase look very similar, implying that the mixing of orbitals may be similar in both phases. Finally, we highlight that one of the Cl^–^ ions in Zn_2_Cl_5_^•2–^ does not lie on the plane of the molecule but instead bridges them, as is depicted in Figure 5.

**Figure 8:**
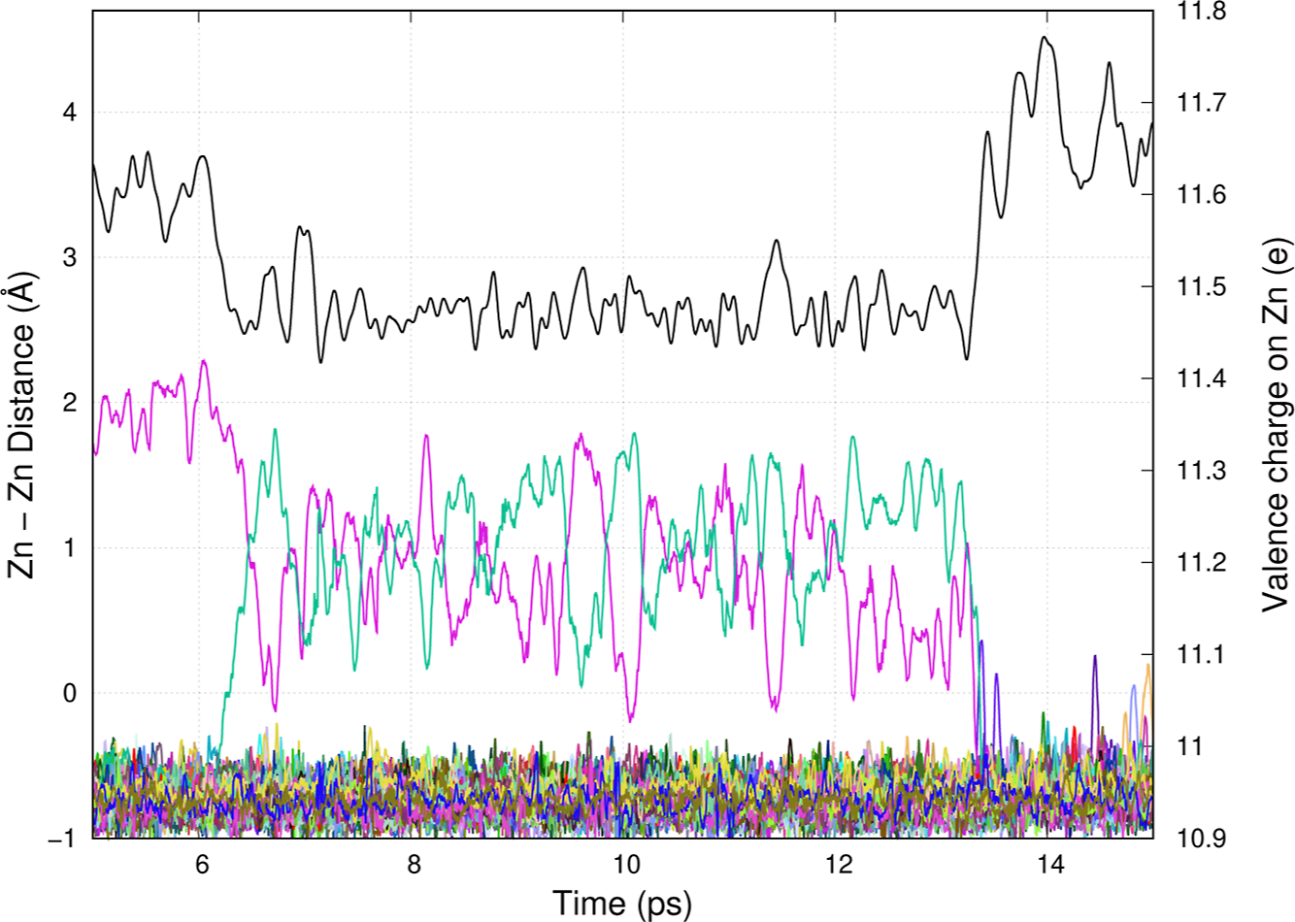
(Color lines) time-dependent valence charge (Bader charges) of Zn ions and (black line) the distance between the two relevant Zn ions that will transform between ZnCl_3_^•2–^ to Zn_2_Cl_5_^•2–^ and the delocalized state.

Before moving on to discuss the spectroscopy of these species, we touch on one more structural aspect. In our recent study,^16^ we found that the first Zn^2+^–Zn^2+^ peak in the pair distribution function *g*(*r*) extends between ≈2.5 Å (where there is no density) to the first minimum in *g*(*r*) at ≈4.5 Å with a maximum at 3.75 Å. Figure 8 shows the distance between two Zn species (black line) and the valence charge of each Zn species (color lines) to visualize what happens as the system goes from ZnCl_3_^•2–^ to Zn_2_Cl_5_^•2–^ to the delocalized state.

It is easiest to look at the time history of the charge starting from longer times to shorter times. At longer times, above 14 ps, the charge is delocalized and the two selected Zn^2+^ ions are at typical distances consistent with the first peak of *g*(*r*) in the ZnCl_2_ melt. Between 7 and 13 ps, the charge is shared between two Zn species forming Zn_2_Cl_5_^•2–^. In this case, the distance is much shorter fluctuating at ≈2.5 Å. Such distances do not exist or have an extremely low probability for Zn^2+^ ions in the neutral melt. In other words, such short distances are indicative of the formation of the Zn_2_Cl_5_^•2–^ species. At shorter times up to 6 ps, the distance between these two Zn species fluctuates in a range that is longer than that observed for Zn_2_Cl_5_^•2–^ but shorter than that which would be typical for first neighbor Zn^2+^ ions in a neutral melt. Such is the distance between ZnCl_3_^•2–^ and an adjacent Zn^2+^ that is about to undergo a chemical reaction to form Zn_2_Cl_5_^•2–^.

### 3.2 Spectra of the Species

In this section, we discuss the optical spectrum of each of the transient species that we have identified. In doing so, several caveats should be considered. First, the spectrum we can compute is that of a single excess e^–^ in the melt, but the experimental spectrum is an ensemble average over many electrons in a macroscopic liquid. It is therefore likely that a spectrum obtained for a single electron in a single simulation snapshot will only provide very qualitative information to contrast against transient spectroscopy. Because of this, we have repeated the calculation multiple times (*vide infra*); doing so using TDDFT and PBE0-D3 is very expensive, even with the considerable computational resources available to the authors. Specifically, we have generated four independent spectra for Zn_2_Cl_5_^•2–^ and ZnCl_3_^•2–^ and two for the delocalized species, none of which are broadened. The idea is to provide a good qualitative idea of what the spectral features may be and how they may fluctuate without attempting to be fully quantitative.

**Figure 9:**
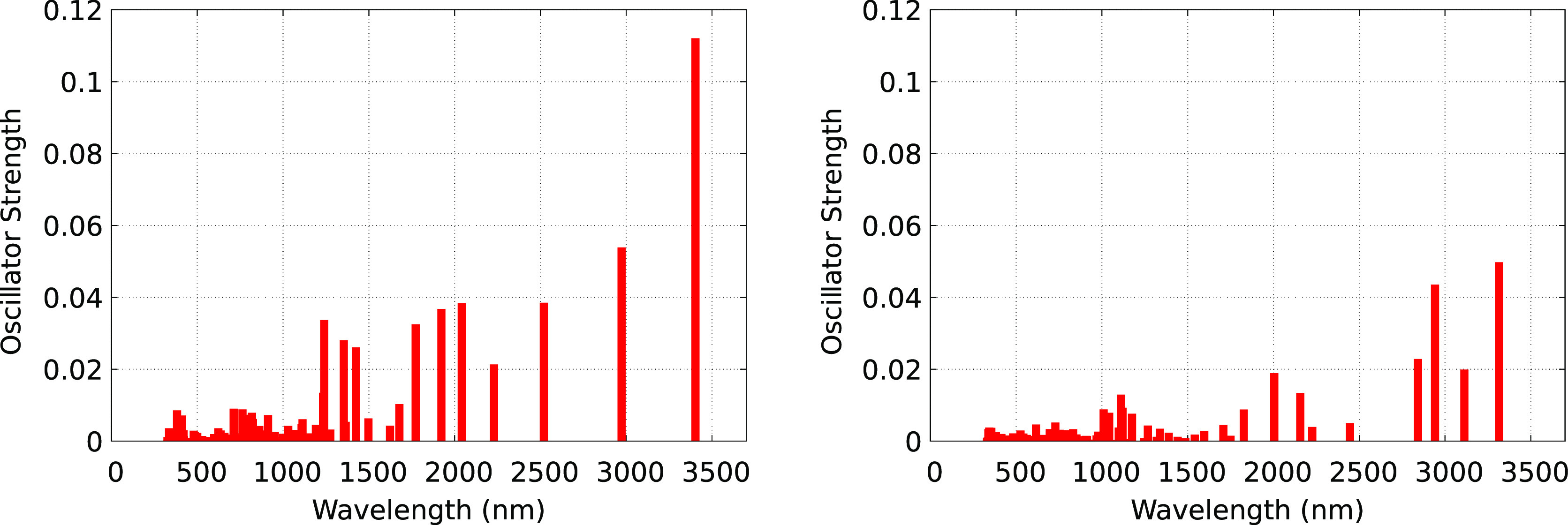
Two independent examples of the excess electron spectrum when it is delocalized using the TDDFT method and PBE0-D3.

Figure 9 shows the spectrum of the excess e^–^ when it is in the delocalized state; this spectrum covers absorption from the visible all the way to the near- and mid-infrared regions. Such a broad spectrum is typical of a dry electron before a suitable Coulombic trap has been found or during delocalization periods between traps (in our case, we are calling ZnCl_3_^•2–^ and Zn_2_Cl_5_^•2–^ traps). Because the energy of SOMO is high when the electron is delocalized, the separation between SOMO and the LUMO band is small; this is the reason for the broad spectrum of the delocalized electron. We have seen such transitions before for ILs^9−12^ and these have been experimentally confirmed for ILs in recent ultrafast spectroscopic studies.^13^

**Figure 10:**
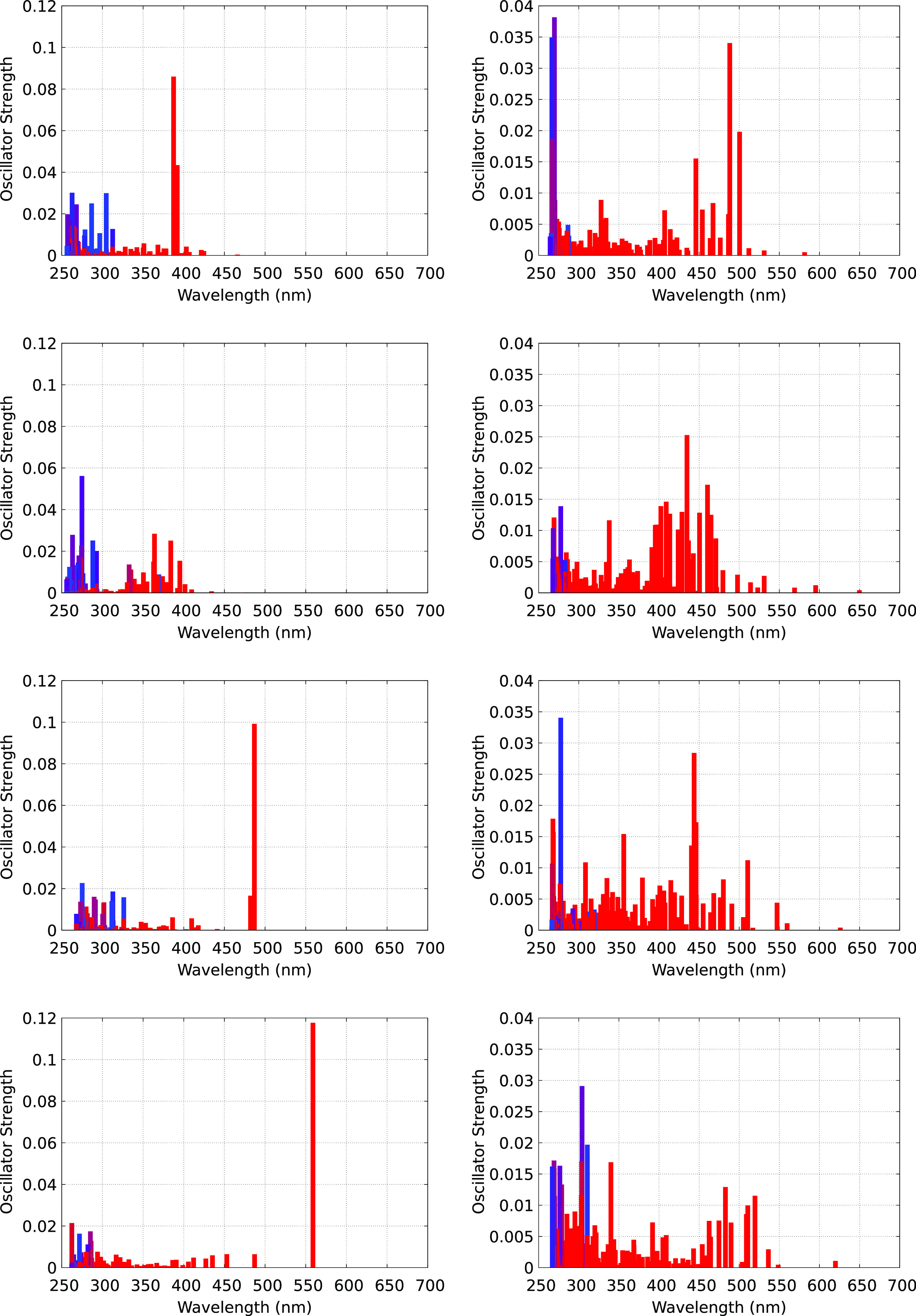
(Left) Four independent spectra where the e^–^ is trapped in the Zn_2_Cl_5_^•2–^ state. Spectra correspond to the snapshots displayed in Figure 5. (Right) Four independent spectra corresponding to the e^–^ in the ZnCl_3_^•2–^ state. All spectra were computed using the TDDFT method and PBE0-D3. The color palette identifies transitions that initiate from SOMO (these are transitions of the excess e^–^) as red and those from the bulk liquid into SOMO or other higher energy states above SOMO as blue. Colors that are in between these imply mixed contributions.

When the electron localizes to form Zn_2_Cl_5_^•2–^ or ZnCl_3_^•2–^, the energetic landscape is very different compared to when it is in the delocalized state, as can be gleaned from Figure 10. We notice that there are no low-energy transitions like in the case of the delocalized e^–^. This is because as we can see in Figure 1, localization results in a significant lowering of the SOMO energy and a concomitant separation between SOMO and the LUMO band (this is particularly the case for Zn_2_Cl_5_^•2–^). Consistent with this, at very low intensity, the spectrum of ZnCl_3_^•2–^ extends all the way to 650 nm, but that of Zn_2_Cl_5_^•2–^ is always below 575 nm. Whereas we are not aware of available experimental assignments of the electron spectrum to different species in the ZnCl_2_ melt, our findings for Zn_2_Cl_5_^•2–^ and ZnCl_3_^•2–^ are quite consistent with those for ZnCl_2_ in the crystal form; see, for example, Figure 9 in ref (43).

We notice that there is a very high-intensity transition for Zn_2_Cl_5_^•2–^ that changes in energy from frame to frame in the range ≈400–550 nm that is not there for ZnCl_3_^•2–^ (spectra on the left and right in Figure 10 have different oscillator strength ranges). We speculate that this high-intensity transition may relate to the particular symmetry of the Zn_2_Cl_5_^•2–^ species as it is absent in the second spectrum that corresponds to the transition situation in Figure 5, where six Cl^–^ share significant electron density with the Zn ions instead of the five in Zn_2_Cl_5_^•2–^. From the color palette in Figure 10, we see that the spectra of both species (Zn_2_Cl_5_^•2–^ and ZnCl_3_^•2–^) include transitions that are not only of the excess e^–^ but also of the neat liquid. These transitions do not exist in the absence of the excess electron but are observed now because (i) the excess electron provides a low-energy (compared to LUMO) SOMO state into which electrons from the bulk can transition and (ii) because the actual LUMO band is different in the presence and absence of the excess electron. In other words, the excess electron introduces liquid-SOMO and liquid-LUMO band transitions where an electron from the bulk liquid can be promoted, leaving a hole behind. Such transitions, which originate from the bulk melt, cannot be subtracted out using a blank sample and are therefore identified as transitions of the excess electron in the interpretation of transient absorption experiments.

## 4 Conclusions

We have studied the short-time fate of an excess electron in ZnCl_2_. We found that a single excess e^–^ can be found as a delocalized species, as ZnCl_3_^•2–^ or as Zn_2_Cl_5_^•2–^. Each of these species has different but very characteristic SOMO energies, orbital composition, and spectra. Interestingly, whereas the energy of SOMO in the case of Zn_2_Cl_5_^•2–^ is significantly lower than that of the other versions of the excess electron, this does not imply that the electron remains trapped in this configuration. Thermal motion in the high-temperature molten salt must preclude this and it would be interesting but beyond current computational means to understand what statistical proportion of each of these species exists in actuality. There is also the question of the so-called “empty volume”; when we run our simulations for a system in which a Cl^–^ ion was removed, localization to form the dimeric form was fast and prominent, and the state was visited again for a large fraction of time. Our simulations in the constant volume ensemble do not allow for expansion upon the introduction of an excess electron. It is likely that, experimentally, local thermal expansion may contribute to the ability of an electron to find a suitable trap. Whereas this may not affect the nature of species present, it may affect the proportion of these in an actual system. Many other fascinating questions remain that are beyond the scope of current computational capabilities, such as the interaction of radicals to form other stable species. We believe that ZnCl_2_ is special in its ability to form “molecular” or covalent radical ion species such as Zn_2_Cl_5_^•2–^ in part (but not only) because it is liquid at a lower temperature than other molten salts. We will be publishing a comparison between ZnCl_2_ and other divalent salt melts in the near future discussing this point.

## Notes

Datasets for this article are made available within 30 days of the official acceptance date of this article by the journal in the Zenodo repository under the Digital Object Identifier (DOI): 10.5281/zenodo.8070875.

## References

[ref1] Forsberg C.; Zheng G. T.; Ballinger R. G.; Lam S. T. Fusion Blankets and Fluoride-Salt-Cooled High-Temperature Reactors with Flibe Salt Coolant: Common Challenges, Tritium Control, and Opportunities for Synergistic Development Strategies Between Fission, Fusion, and Solar Salt Technologies. Nucl. Technol. 2019, 206, 1778–1801. 10.1080/00295450.2019.1691400.

[ref2] Forsberg C. W. Market Basis for Salt-Cooled Reactors: Dispatchable Heat, Hydrogen, and Electricity with Assured Peak Power Capacity. Nucl. Technol. 2020, 206, 1659–1685. 10.1080/00295450.2020.1743628.

[ref3] Odenthal C.; Klasing F.; Knödler P.; Zunft S.; Bauer T. Experimental and numerical investigation of a 4 MWh high temperature molten salt thermocline storage system with filler. AIP Conf. Proc. 2020, 2303, 190025 10.1063/5.0028494.

[ref4] Andreades C.; Cisneros A. T.; Choi J. K.; Chong A. Y. K.; Fratoni M.; Hong S.; Huddar L. R.; Huff K. D.; Kendrick J.; Krumwiede D. L.; et al. Design Summary of the Mark-I Pebble-Bed, Fluoride Salt–Cooled, High-Temperature Reactor Commercial Power Plant. Nucl. Technol. 2016, 195, 223–238. 10.13182/nt16-2.

[ref5] Grimes W. R. Chemical, Research and Development for Molten-Salt Breeder Reactors; ORNL-TM-1853, 1967.

[ref6] Iwamatsu K.; Horne G. P.; Gakhar R.; Halstenberg P.; Layne B.; Pimblott S. M.; Wishart J. F. Radiation-induced reaction kinetics of Zn^2+^ with e_S_ and Cl2• in Molten LiCl–KCl eutectic at 400–600 ^°^C. Phys. Chem. Chem. Phys. 2022, 24, 25088–25098. 10.1039/d2cp01194h. 35789354

[ref7] Hagiwara H.; Sawamura S.; Sumiyoshi T.; Katayama M. Pulse radiolysis study of transient species in LiCl-KCl melt. Radiat. Phys. Chem. 1987, 30, 141–144. 10.1016/1359-0197(87)90097-x.

[ref8] Sawamura S.; Gȩbicki J.; Mayer J.; Kroh J. Pulse radiolysis of LiBr-KBr melts. Optical transient absorption spectra. Radiat. Phys. Chem. 1990, 36, 133–136. 10.1016/1359-0197(90)90228-a.

[ref9] Margulis C. J.; Annapureddy H. V. R.; De Biase P. M.; Coker D.; Kohanoff J.; Del Pópolo M. G. Dry Excess Electrons in Room-Temperature Ionic Liquids. J. Am. Chem. Soc. 2011, 133, 20186–20193. 10.1021/ja203412v. 22032301

[ref10] Xu C.; Durumeric A.; Kashyap H. K.; Kohanoff J.; Margulis C. J. Dynamics of Excess Electronic Charge in Aliphatic Ionic Liquids Containing the Bis(trifluoromethylsulfonyl)amide Anion. J. Am. Chem. Soc. 2013, 135, 17528–17536. 10.1021/ja409338z. 24156701

[ref11] Xu C.; Margulis C. J. Solvation of an Excess Electron in Pyrrolidinium Dicyanamide Based Ionic Liquids. J. Phys. Chem. B 2015, 119, 532–542. 10.1021/jp5108922. 25523330

[ref12] Dhungana K. B.; Wu F.; Margulis C. J. Excess Electron and Hole in 1-Benzylpyridinium-Based Ionic Liquids. J. Phys. Chem. B 2017, 121, 8809–8816. 10.1021/acs.jpcb.7b07499. 28846846

[ref13] Knudtzon M. N.; Blank D. A. Photodetachment and Electron Dynamics in 1-Butyl-1-methyl-pyrrolidinium Dicyanamide. J. Phys. Chem. B 2020, 124, 9144–9153. 10.1021/acs.jpcb.0c06508. 32955885

[ref14] Causo M. S.; Ciccotti G.; Montemayor D.; Bonella S.; Coker D. F. An Adiabatic Linearized Path Integral Approach for Quantum Time Correlation Functions: Electronic Transport in Metal-Molten Salt Solutions. J. Phys. Chem. B 2005, 109, 6855–6865. 10.1021/jp045208b. 16851772

[ref15] Selloni A.; Carnevali P.; Car R.; Parrinello M. Localization, hopping, and diffusion of electrons in molten salts. Phys. Rev. Lett. 1987, 59, 823–826. 10.1103/physrevlett.59.823. 10035880

[ref16] Roy S.; Brehm M.; Sharma S.; Wu F.; Maltsev D. S.; Halstenberg P.; Gallington L. C.; Mahurin S. M.; Dai S.; Ivanov A. S.; et al. Unraveling Local Structure of Molten Salts via X-ray Scattering, Raman Spectroscopy, and *Ab Initio* Molecular Dynamics. J. Phys. Chem. B 2021, 125, 5971–5982. 10.1021/acs.jpcb.1c03786. 34037400

[ref17] Lucas P.; Coleman G. J.; Venkateswara Rao M.; Edwards A. N.; Devaadithya C.; Wei S.; Alsayoud A. Q.; Potter B. G.; Muralidharan K.; Deymier P. A. Structure of ZnCl_2_ Melt. Part II: Fragile-to-Strong Transition in a Tetrahedral Liquid. J. Phys. Chem. B 2017, 121, 11210–11218. 10.1021/acs.jpcb.7b10857. 29166015

[ref18] Wu F.; Roy S.; Ivanov A. S.; Gill S. K.; Topsakal M.; Dooryhee E.; Abeykoon M.; Kwon G.; Gallington L. C.; Halstenberg P.; et al. Elucidating Ionic Correlations Beyond Simple Charge Alternation in Molten MgCl_2_-KCl Mixtures. J. Phys. Chem. Lett. 2019, 10, 7603–7610. 10.1021/acs.jpclett.9b02845. 31738562

[ref19] Sharma S.; Emerson M. S.; Wu F.; Wang H.; Maginn E. J.; Margulis C. J. SEM-Drude Model for the Accurate and Efficient Simulation of MgCl_2_-KCl Mixtures in the Condensed Phase. J. Phys. Chem. A 2020, 124, 7832–7842. 10.1021/acs.jpca.0c06721. 32841019

[ref20] Wu F.; Sharma S.; Roy S.; Halstenberg P.; Gallington L. C.; Mahurin S. M.; Dai S.; Bryantsev V. S.; Ivanov A. S.; Margulis C. J. Temperature Dependence of Short and Intermediate Range Order in Molten MgCl_2_ and Its Mixture with KCl. J. Phys. Chem. B 2020, 124, 2892–2899. 10.1021/acs.jpcb.0c00745. 32148043

[ref21] Dias E. T.; Gill S. K.; Liu Y.; Halstenberg P.; Dai S.; Huang J.; Mausz J.; Gakhar R.; Phillips W. C.; Mahurin S.; et al. Radiation-Assisted Formation of Metal Nanoparticles in Molten Salts. J. Phys. Chem. Lett. 2020, 12, 157–164. 10.1021/acs.jpclett.0c03231. 33320682

[ref22] Hutter J.; Iannuzzi M.; Schiffmann F.; VandeVondele J. CP2K: atomistic simulations of condensed matter systems. Wiley Interdiscip. Rev.: Comput. Mol. Sci. 2014, 4, 15–25. 10.1002/wcms.1159.

[ref23] Kühne T. D.; Iannuzzi M.; Del Ben M.; Rybkin V. V.; Seewald P.; Stein F.; Laino T.; Khaliullin R. Z.; Schutt O.; Schiffmann F.; et al. CP2K: An electronic structure and molecular dynamics software package - Quickstep: Efficient and accurate electronic structure calculations. J. Chem. Phys. 2020, 152, 194103 10.1063/5.0007045. 33687235

[ref24] Perdew J. P.; Burke K.; Ernzerhof M. Generalized gradient approximation made simple. Phys. Rev. Lett. 1996, 77, 3865–3868. 10.1103/physrevlett.77.3865. 10062328

[ref25] Grimme S.; Antony J.; Ehrlich S.; Krieg H. A consistent and accurate ab initio parametrization of density functional dispersion correction (DFT-D) for the 94 elements H-Pu. J. Chem. Phys. 2010, 132, 154104 10.1063/1.3382344. 20423165

[ref26] VandeVondele J.; Hutter J. Gaussian basis sets for accurate calculations on molecular systems in gas and condensed phases. J. Chem. Phys. 2007, 127, 114105 10.1063/1.2770708. 17887826

[ref27] Goedecker S.; Teter M.; Hutter J. Separable dual-space Gaussian pseudopotentials. Phys. Rev. B: Condens. Matter Mater. Phys. 1996, 54, 1703–1710. 10.1103/physrevb.54.1703. 9986014

[ref28] VandeVondele J.; Hutter J. An efficient orbital transformation method for electronic structure calculations. J. Chem. Phys. 2003, 118, 4365–4369. 10.1063/1.1543154.

[ref29] Martyna G. J.; Klein M. L.; Tuckerman M. Nosé–Hoover chains: The canonical ensemble via continuous dynamics. J. Chem. Phys. 1992, 97, 2635–2643. 10.1063/1.463940.

[ref30] Nosé S. A unified formulation of the constant temperature molecular dynamics methods. J. Chem. Phys. 1984, 81, 511–519. 10.1063/1.447334.

[ref31] Sanville E.; Kenny S. D.; Smith R.; Henkelman G. Improved grid-based algorithm for Bader charge allocation. J. Comput. Chem. 2007, 28, 899–908. 10.1002/jcc.20575. 17238168

[ref32] Tang W.; Sanville E.; Henkelman G. A grid-based Bader analysis algorithm without lattice bias. J. Phys.: Condens. Matter 2009, 21, 084204 10.1088/0953-8984/21/8/084204. 21817356

[ref33] Becke A. D. Density-Functional Thermochemistry .3. The Role of Exact Exchange. J. Chem. Phys. 1993, 98, 5648–5652. 10.1063/1.464913.

[ref34] Lee C. T.; Yang W. T.; Parr R. G. Development of the Colle-Salvetti Correlation-Energy Formula into a Functional of the Electron-Density. Phys. Rev. B: Condens. Matter Mater. Phys. 1988, 37, 785–789. 10.1103/physrevb.37.785. 9944570

[ref35] Vosko S. H.; Wilk L.; Nusair M. Accurate spin-dependent electron liquid correlation energies for local spin density calculations: a critical analysis. Can. J. Phys. 1980, 58, 1200–1211. 10.1139/p80-159.

[ref36] Stephens P. J.; Devlin F. J.; Chabalowski C. F.; Frisch M. J. Ab Initio Calculation of Vibrational Absorption and Circular Dichroism Spectra Using Density Functional Force Fields. J. Phys. Chem. 1994, 98, 11623–11627. 10.1021/j100096a001.

[ref37] Weigend F.; Ahlrichs R. Balanced basis sets of split valence, triple zeta valence and quadruple zeta valence quality for H to Rn: Design and assessment of accuracy. Phys. Chem. Chem. Phys. 2005, 7, 3297 10.1039/b508541a. 16240044

[ref38] Frisch M. J.; Trucks G. W.; Schlegel H. B.; Scuseria G. E.; Robb M. A.; Cheeseman J. R.; Scalmani G.; Barone V.; Mennucci B.; Petersson G. A.; Gaussian 09, Revision D.01; Gaussian Inc.: Wallingford CT, 2009.

[ref39] Hehn A.-S.; Sertcan B.; Belleflamme F.; Chulkov S. K.; Watkins M. B.; Hutter J. Excited-State Properties for Extended Systems: Efficient Hybrid Density Functional Methods. J. Chem. Theory Comput. 2022, 18, 4186–4202. 10.1021/acs.jctc.2c00144. 35759470 PMC9281608

[ref40] Adamo C.; Barone V. Toward reliable density functional methods without adjustable parameters: The PBE0 model. J. Chem. Phys. 1999, 110, 6158–6170. 10.1063/1.478522.

[ref41] Guidon M.; Hutter J.; VandeVondele J. Auxiliary Density Matrix Methods for Hartree-Fock Exchange Calculations. J. Chem. Theory Comput. 2010, 6, 2348–2364. 10.1021/ct1002225. 26613491

[ref42] Glendening E. D.; Reed A. E.; Carpenter J. E.; Weinhold F. NBO Program, Version 3.1; Gaussian Inc., 2003.

[ref43] Ramos-Ballesteros A.; Gakhar R.; Horne G. P.; Iwamatsu K.; Wishart J. F.; Pimblott S. M.; LaVerne J. A. Gamma radiation-induced defects in KCl, MgCl_2_, and ZnCl_2_ salts at room temperature. Phys. Chem. Chem. Phys. 2021, 23, 10384–10394. 10.1039/d1cp00520k. 33889900

